# Supplementary material for: Clinical application of HEDI for biomechanical evaluation and visualisation in incisional hernia repair
Source: Commun Med (Lond). 2026 Jan 4;6:68. doi: 10.1038/s43856-025-01311-w (PMC12864906; doi:10.1038/s43856-025-01311-w)
Supplement: Supplementary file 2 — Description of Additional Supplementary Data [file 43856_2025_1311_MOESM2_ESM.docx]

Description of additional supplementary file

File name: Supplementary Data

Description: The raw pain score data from 31 patients undergoing incisional hernia repair, which form the basis of Fig. 3, are provided as Supplementary Data with this manuscript.
